# The Bombing of Bristol

**Published:** 1940

**Authors:** 


### THE BOMBING OF BRISTOL.

THE first air attack on Bristol came in July and by night. Few bombs were dropped, one just missing the Eye Hospital and demolishing the house next it. Brislington sustained damage and some casualties. Desultory attacks followed, two or three "alerts" every day or night being usual, but the damage was insignificant.

In September, a mid-day mass attack was delivered. No serious damage was done, but there were a number of casualties. The raid revealed defects both in the arrangements for treating casualties on the spot, and in the methods of transport adopted. Adequate preparations for an incident of this kind require a medical director of experience, with good organizing ability and the capacity for getting done what he considers essential. A similar attack, two days later, was broken up by our fighters, one of the attacking machines being shot down in the grounds of Fishponds Mental Hospital.

One Sunday in November, a very heavy night attack was delivered on Bristol itself, many hundreds of incendiary bombs being followed by successive waves of high explosive. Large fires were started. Up to the time of going to press there have been several such attacks. It would be idle to deny that considerable material damage has been done in the centre of Bristol, and some of the main shopping thoroughfares, as well as many streets of small houses. Fortunately the first attack was on a Sunday when the streets were comparatively empty. Casualties have been astonishingly light considering the intensity of the raids, and the E.M.S. organization operated without a hitch.

As recently as last May, the date for the transfer of departments consequent on the amalgamation of the Royal Infirmary and the General Hospital was discussed: a "minority" favoured postponing this change, the contention being: (1) in any case a large re-organization will be necessary after the war; (2) during and immediately after any heavy attack communications by the bridges and narrow streets in the centre of Bristol must be precarious; (3) it is unwise to assemble the entire equipment of any one department where a single unlucky bomb could destroy the whole; and (4) the General Hospital building amidst factories and warehouses, and surrounded by river, dock, railway and wharves is "strategically" very bad. But ἡ Ἀνάγκη prevailed. In the second big raid most of the windows of the Hospital were broken and doors were blown in. The Children's Hospital had a similar experience. When incendiaries fell on another hospital very little damage was done owing to the excellent "fire-fighting" by staff and patients. Thanks to the untiring and undaunted efforts of staffs and ambulance drivers the removal of patients from various institutions was carried out during the hottest period of raids without casualty.

The University Great Hall has been gutted, and therein the library of the Faculty of Theology from King's College: and among other damage the whole of the Anatomy building and its contents were destroyed. Fortunately the University libraries, including the Medical, escaped, and it is hoped to distribute the valuable books in less dangerous areas. St. Peter's, with its quaint carvings, the first Poor-Law Hospital in the country (1696–1852), has perished.

The destruction of large drug houses in the centre of the city has hampered professional work. Very many citizens whose houses were intact, or at most without window glass, found that the cutting off of water supplies was a real nuisance. Not only had water to be fetched in jugs and boiled before use: but the sanitary arrangements of a modern house are dependent on ample water supply. Fortunately the warnings issued by the public health department were effective, and water-borne infection has been avoided. When mains and sewers are broken in the same crater the risk of contamination must be considerable.

The E.M.S. arrangements for such a situation have been brought into operation. A general hospital has been opened, the patients being taken to and from Bristol by ambulance; it is hoped to obtain additional accommodation shortly. The General Hospital has been repaired. The work of the out-patient departments has never been interrupted.

We have learnt several valuable lessons from these raids. First and foremost, the damage from even the heaviest high explosive bomb is usually very local. "Blast" injuries have not been troublesome, and those under cover have little to fear unless the bomb falls exactly where they are sheltering. The second lesson is how very crippling to hospital work is interference with roads, telephones, gas, above all water and electricity: it is essential to make provision for the work to be carried out well away from areas that are likely targets for intensive bombing. In the third place, as experience has demonstrated (and as a group of them has pointed out), far greater use could be made in first-aid work of the doctors on the spot, or in the vicinity of incidents—if they are provided with the essential equipment.* It is unsound to rely exclusively on help being despatched from a central depot, for during an intensive raid many streets may become simply impassable. Finally the population has stood up well to the physical and nervous strain. There have been very few cases of neurosis; and mental disease shows an actual decrease. Bristol is being tidied up and work is being resumed after an amazingly short delay.

Our Society, however, overtaken at the declaration of war by a profound lethargy from which it seemed about to emerge, has sunk back into a completely comatose condition.

* Since these notes were printed, March 1st, arrangements have been made to remedy this defect.

